# β‐Glucan supplementation, allergy symptoms, and quality of life in self‐described ragweed allergy sufferers

**DOI:** 10.1002/fsn3.11

**Published:** 2014-01-08

**Authors:** Shawn M Talbott, Julie A Talbott, Tracy L Talbott, Elaine Dingler

**Affiliations:** GLH NutritionLLC, Draper, UT, 84020

**Keywords:** Allergy, allergy symptom, β-glucan, IgE, quality of life, ragweed, rhinitis

## Abstract

This randomized, placebo-controlled, double-blind study compared the effects of daily supplementation for 4 week with 250 mg Wellmune WGP® β-1,3/1,6-Glucan (WGP) with placebo 250 mg/day (rice flour) on physical and psychological health attributes of self-described “moderate” ragweed allergy sufferers. Study participants (mean age = 36 ± 9 year; range 18–53 year) were recruited before the beginning of ragweed season (September) in Northeastern Ohio. Serum IgE concentration, allergy symptoms [via self-report, Visual Analog Scale (VAS), and Rhinoconjunctivitis Quality of Life Questionnaire (RQLQ)], psychological well-being [Profile of Mood States (POMS)], and physical function (RAND SF-36 Medical Outcomes Study) were measured immediately prior to and after supplementation with WGP (*n = *24) or placebo (*n = *24) for 4 weeks. Data were analyzed using repeated measures analyses of variance (ANOVA). Compared with placebo, WGP reduced total allergy symptoms (28%), symptom severity (52%), and symptom rating on the VAS (37%) (*P *<* *0.05), but had no effect on serum IgE levels. As measured by the POMS, WGP increased participants' rating of vigor (10%), but reduced tension (34%), depression (45%), anger (41%), fatigue (38%), and confusion (34%) (*P *<* *0.05). Study participants given WGP reported increased physical health (11%), energy (19%), and emotional well-being (7%) compared with study participants given the placebo (RAND SF-36 Medical Outcomes Study). The WGP group also reported decreased sleep problems (53%), reduced nasal symptoms (59%), eye symptoms (57%), non-nasal symptoms (50%), activities (53%), emotions (57%), and improved quality of life (QOL) (56%), as well as improved global mood state (13%). Supplementation with WGP for 4 weeks improved allergy symptoms, overall physical health, and emotional well-being compared with placebo in self-described “moderate” ragweed allergy sufferers during ragweed allergy season.

## Introduction

An estimated 40–50 million Americans are affected by allergic disease (National Institute of Allergy and Infectious Diseases 2003). Pollen from plants of the genus Ambrosia (which includes ragweed) is a primary cause of allergic rhinitis, also known as hay fever, during summer and fall (Frenz et al. 1995). In the U.S., ragweed season can extend from August through October (Pongdee 2011). The U.S. National Health and Nutrition Examination Survey III conducted from 1988 through 1994 reported a prevalence of 26.2% for ragweed allergy as determined by skin test reactivity (Arbes et al. 2005). Approximately 45% of atopic individuals in the U.S. and Canada are allergic to ragweed (Boulet et al. 1997) and the prevalence of allergy to ragweed is increasing in some areas of Europe (Zanon et al. 2002; Wopfner et al. 2005; Oswalt and Marshall 2008).

Allergic disease has various manifestations. Seasonal allergic rhinitis is an IgE-mediated inflammatory disease of the nasal mucous membranes that often presents with rhinorrhea, sneezing, nasal congestion, and itching, as well as ocular redness, tearing, and itching (Nathan 2003). Allergic rhinitis is associated with reduced quality of life (QOL) (Leynaert et al. 2000; Meltzer 2001), and decreased work productivity, accounting for an estimated 3.5 million work days and 2 million school days lost per year (Nathan 2007). In addition to local inflammation, allergic rhinitis can induce systemic inflammation, which may result in inflammation of the upper and lower airways (Pawankar et al. 2011). Comorbid conditions for allergic rhinitis include asthma, rhinosinusitis, nasal polyposis, serous otitis media, and sleep disorders (Young 1998; Bousquet et al. 2001; Pawankar et al. 2011). Allergic conjunctivitis affects more than 20% of the U.S. population (Nathan 2003) and approximately 18% of children, aged 12–14 in the U.K. (Austin et al. 1999). Inadequate control of allergic rhinoconjunctivitis leads to decreased QOL, reduced productivity, and potentially, increased visits to doctor's offices.

Approaches to reduce the physical and psychological symptoms of allergic disease include allergen avoidance, pharmacotherapy, and immunotherapy (Bousquet et al. 1994). Standard allergen immunotherapy can prove problematic due to inconvenient dosing regimens and the potential for induction of systemic allergic reactions, such as anaphylaxis, because of the relatively large doses of allergen administered (Lockey et al. 1987; Du Buske et al. 1992; Bukantz and Lockey 2004). A safe, effective, and convenient therapy for allergic disease would provide relief of the physical and psychological effects of allergies and improve the QOL of allergy sufferers (Kim et al. 2011).

β-Glucans are a diverse class of long-chain glucose polymers that have a backbone of β-(1-3)-linked β-d-glucopyranosyl units with primarily β-(1,4)-or (1,6)-linked side chains. These naturally occurring substances have been shown to provide health benefits including wound healing (Delatte et al. 2001), decreasing blood lipid concentration (Bell et al. 1999; Nicolosi et al. 1999), protection against infection (de Felippe 1993; Babineau et al.^1994^a,b; Dellinger et al. 1999), inhibition of tumor development (Cheung et al. 2002; Hong et al. 2004) and metastasis (Yoon et al. 2008), and promotion of tumor regression (Driscoll et al. 2009). β-Glucans are known as biological response modifiers. Their effects have been investigated by administration through diverse routes; importantly, they exhibit biological activity when consumed orally (Hong et al. 2004; Rice et al. 2005) and may be combined with other immunotherapies to elicit a more powerful effect (Hong et al. 2004; Chen and Seviour 2007). They activate the entire immune system, which may be advantageous for their use as therapy for disease.

β-Glucans are found abundantly in bacterial and fungal cell walls and in plants, such as oat, barley, and seaweed (Akramiene et al. 2007). β-Glucans derived from different sources exhibit structural differences and their biological activities are determined by molecular structure, size, degree of branching, structural modification, conformation, and solubility (Kim et al. 2011). Because mammalian cells do not synthesize β-glucans, they are recognized as pathogen-associated molecular patterns (PAMPs) by germline-encoded pattern recognition receptors (PRRs) on cell surfaces (Willment et al. 2005). PRRs that recognize β-glucans include the toll-like receptors (TLR), C-type lectin receptors (CLR) such as dectin-1 (Brown and Gordon 2001; Brown et al. 2002, 2003; Taylor et al. 2002; Tsoni and Brown 2008), complement receptor 3 (CR3) (Ross et al. 1987; Thornton et al. 1996), lactosylceramide (Zimmerman et al. 1998), and scavenger receptors (Pearson et al. 1995; Rice et al. 2002). β-Glucan receptors have been identified on the surfaces of immune cells, including neutrophils, eosinophils, natural killer cells, endothelial cells, alveolar epithelial cells, fibroblasts, and various types of macrophages (Brown 2006; Shah et al. 2008), as well as nonimmune cells, such as epithelial cells (Ahren et al. 2001), vascular endothelial cells (Lowe et al. 2002), and fibroblasts (Kougias et al. 2001). β-Glucans are powerful activators of macrophages/monocytes (Adachi et al. 1994; Lebron et al. 2003) and neutrophils (Zhang and Petty 1994) and are primarily responsible for stimulating the reticuloendothelial system (Di Carlo and Fiore 1958; Riggi and Di Luzio 1961; Wooles and Diluzio 1963).

Identification of β-glucans as nonself molecules by mammalian cells induces innate and adaptive immune responses (Vetvicka and Yvin 2004; Brown and Gordon 2005). The initial response to β-glucans occurs via the innate immune system. This response is rapid and nonspecific, occurs without memory, and primarily involves phagocytic cells, such as macrophages and neutrophils. Following ingestion, β-glucans are bound to PRRs on macrophages and are internalized. As reviewed by Volman et al. (2008), β-glucans interact with the mucosal immune system including the intestinal intraepithelial lymphocytes and Peyer's patches, inducing cytokine production and increasing resistance to infection (Suzuki et al. 1990; Tsukada et al. 2003). Whole glucan particle (WGP), a purified yeast β-glucan sphere derived from standard food-grade Baker's yeast, *Saccharomyces cerevisiae,* enters the gut and is taken up by Microfold (M) cells in the Peyer's patches. Intestinal macrophages transport β-glucan to the organs of the immune system (e.g., the spleen, lymph nodes, and bone marrow) via the lymphatic system In the bone marrow, larger WGP are degraded into smaller fragments by macrophages (Hong et al. 2004). β-Glucan-induced signaling is not dependent on internalization of whole β-glucan particles (McCann et al. 2005). β-Glucans are believed to induce phagocytosis (Ladanyi et al. 1993; Kurashige et al. 1997; Brown et al. 2002), stimulate microbial killing by mechanisms such as respiratory burst (Tsoni and Brown 2008), and initiate production of innate immune system components including inflammatory mediators such as tumor necrosis factor alpha (TNF-α), interleukin-1 (IL-1) (Li et al. 2006), macrophage inflammatory protein 2-alpha (MIP-2), eicosanoids, reactive oxidants (Vassallo et al. 1999, 2000; Hahn et al. 2003), and local immunomodulators. Effects of β-glucans on nonimmune cells have also been reported (Hahn et al. 2003; Ramakers et al. 2007). Other cells are recruited to the infection site and activate the adaptive immune system.

The response of the adaptive immune system involves production of antibodies against specific substances by B-lymphocytes. Assay of IgE can determine whether an individual has been sensitized to a substance such as ragweed. The adaptive immune system response also involves antigen presentation by leukocytes to T-lymphocytes. Cytotoxic and T-helper cells attack infected body cells (Volman et al. 2008) and depending on the antigen presented, results in the development of immature cells to type 1 helper T-lymphocytes (Th1) or type II helper T-lymphocytes (Th2) (Volman et al. 2008). Individuals experiencing an allergic response of asthma are thought to have an overactive Th2 response. β-1,3-glucan can stimulate macrophages, which secrete anti-inflammatory mediators, such as prostaglandin E2, tumor growth factor, and IL-10, and may inhibit the Th2 response (Sarinho et al. 2009).

In this study, we investigated whether supplementation with WGP would alleviate the physical and psychological effects of allergies and improve QOL in self-described “moderate” ragweed allergy sufferers during ragweed allergy season.

## Materials and Methods

### Recruitment of study participants

**Figure 1:**
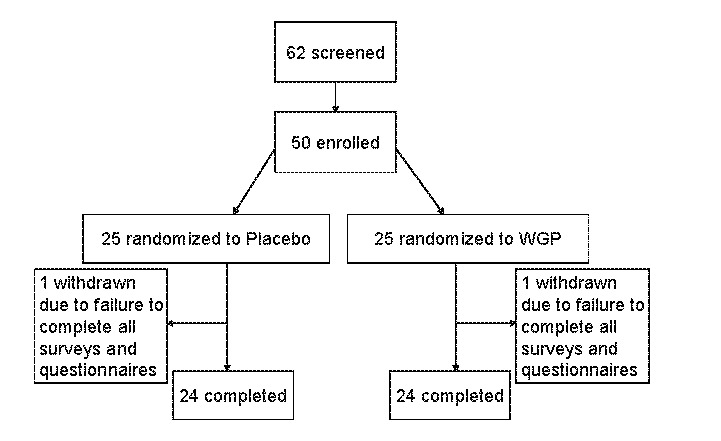
Flow of study participants

The conduct of this study adhered to the Helsinki Declaration, which was revised in 1983, for clinical research involving humans. To recruit subjects, we posted flyers at a walk-in medical clinic where we have conducted previous studies, asking that “people with ragweed allergies” contact us. We screened 62 otherwise healthy self-described “moderate” sufferers of ragweed allergy or individuals who considered themselves to have “hay fever” before peak ragweed allergy season in Northeastern Ohio in 2010 during September and October. Subjects were given a description of the study protocol. On the first visit, we conducted a physical examination of subjects. Individuals who had current URTI symptoms, were currently taking allergy or asthma medications, cited an inability to understand the study objective, were unable to complete all questionnaires and provide blood samples, were pregnant or lactating, or were currently using antibiotics or other “immune” support products were excluded from the study. The Institutional Review Board at Aspire IRB, La Mesa, CA, approved the protocol for the study. All study participants provided written informed consent prior to the start of the study. The flow of the study participants is shown in Figure 1 and demographic information about the study population is shown in Table 1.

**Table 1:** Subject characteristics (baseline)

Treatment group	Placebo	WGP 3-6 β-glucan
*N*	24	24
Age		
Mean ± SD	39 ±9	36 ± 11
Range	18–66 years	18–65 years
Sex		
Men	5 (20%)	12 (50%)
Female	19 (80%)	12 (50%)

*N,* number of subjects; all subjects were self-described moderate ragweed allergy sufferers.

### Wellmune WGP® β-1,3/1,6-glucan

The source of Wellmune WGP® β-1,3/1,6-glucan (WGP) was a purified proprietary strain of Baker's yeast (*Saccharomyces cerevisiae*). WGP was supplied by Biothera, The Immune Company (Eagan, MN). Wellmune WGP is generally recognized as safe (GRAS) (U.S. Food and Drug Administration 2008).

### Study design and intervention

We conducted a randomized, placebo-controlled, double-blind study. Study participants were randomly assigned using a random number generator to one of two treatment groups and were instructed to ingest a capsule containing 250 mg WGP (WGP group) or 250 mg of rice flour (placebo group) once per day, preferably in the morning for 4 weeks. Fifty of the 62 individuals that we screened met the inclusion criteria for the study, and 25 study participants were assigned to each treatment group. Study participants reported to the test site 7 days prior to the start of the study. We supplied study participants with sufficient WGP and placebo capsules to last for the duration of the study. One subject from each group did not complete the study. The final subject population consisted of 17 men and 31 women (36 ± 9; range 18–53 years of age) (see Table 1).

### Outcome measures

The outcomes of the study were ragweed-specific blood IgE concentrations and physical and psychological health attributes of the study participants. Measurements were made at baseline and after study participants consumed WGP or placebo for 4 weeks. IgE concentrations were measured using stored serum samples (*N = *48). Measurements were made for three common ragweed allergens (Giant, Common, and False). IgE levels were measured using the IMMULITE 2000 3gAllergy test kit (Siemens AG, Eschborn, Germany; as described by Biagini et al. 2006). Subjects were asked to complete study surveys that provided information about their physical symptoms (allergy symptoms via self-report, the Visual Analog Scale [VAS; Hornblow and Kidson 1976; Price et al. 1983; Linder 1988; Hallen et al. 2001; Bousquet et al. 2007]; and Rhinoconjunctivitis Quality of Life Questionnaire [RQLQ; Juniper and Guyatt 1991; Juniper et al. 1996]); psychological well-being (Profile of Mood States [POMS; McNair et al. 1971, 2003]); global mood state (POMS); and physical function (RAND SF-36 Medical Outcomes Survey [Ware 1992; Hays et al. 1993]).

### Compliance and adverse events

Compliance with the study protocol was determined by completion and return of study surveys and verbal compliance to daily supplementation instruction. Pills were counted when subjects returned their bottles of capsules at the last visit. Except for two individuals, study participants were better than 90% compliant with the study protocol. The two subjects who did not comply with the study protocol were dropped from the study. Adverse-event information was collected by verbal disclosure; no adverse events (other than allergy symptoms, the primary outcome of the study design) were reported in this study.

### Statistical analysis

Data were analyzed using repeated measures analyses of variance (ANOVA). Statistical analysis was conducted using JMP 8.0 (SAS Institute, Cary, NC) software and significance was set at *P *<* *0.05. The study was set up as a pilot study to determine design criteria for future rhinoconjunctivitis studies; no power calculations were performed.

## Results

Fifty subjects were enrolled in the study; 48 individuals completed the study. Subject attrition, one from each treatment group, was due to failure to submit all completed surveys and questionnaires.

### Physical Symptoms (Allergy Symptoms and RQLQ Survey)

**Figure 2:**
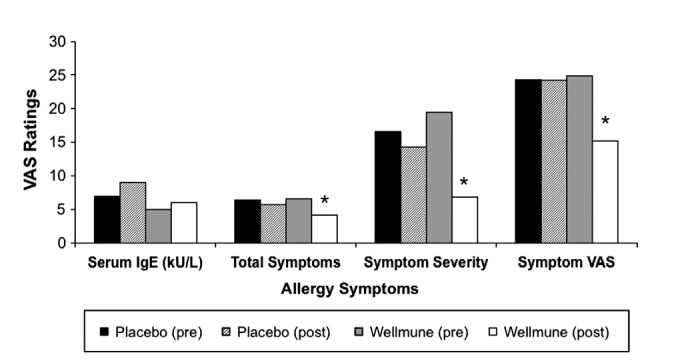
Allergy Symptoms. When compared with placebo treatment with Wellmune WGP® for 4 weeks, there was a reduction observed in total number of allergy symptoms (28%), in symptom severity (52%),and in the rating of symptoms on the VAS (37%).*P <* *0.05 compared with placebo.

Figure 2 compares the serum IgE concentrations (reported in kU/L), total number of allergy symptoms, allergy symptom severity, and allergy symptom scores on the VAS (Hornblow and Kidson 1976; Price et al. 1983; Linder 1988; Hallen et al. 2001; Bousquet et al. 2007) before and 4 weeks after treatment with WGP or placebo. There were no differences in ragweed-specific serum IgE levels. Compared with the placebo post-treatment group, the total number of allergy symptoms (Sx) for the WGP group was reduced 28% (WGP group post-treatment = 4.2, placebo group post-treatment = 5.8, *P *<* *0.001), and participants' ratings of symptom severity were reduced 52% (WGP group post-treatment = 6.9, placebo group post-treatment = 14.3, *P *<* *0.001). The rating of symptoms on the VAS was reduced by 37% after study participants were treated with WGP compared with after treatment with placebo for 4 weeks (WGP group post-treatment = 15.2 vs. placebo group post-treatment = 24.2, *P *=* *0.024).

#### Profile of mood states

**Figure 3:**
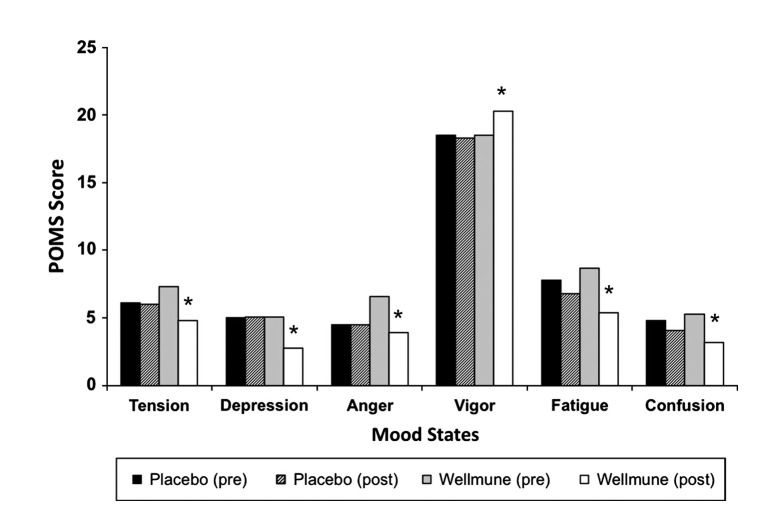
Profile of Mood States. POMS factors improved from week 0 to week 4 following treatment with Wellmune WGP® including reduced tension (34%), depression (45%), anger (41%), fatigue (38%), confusion (34%), and increased vigor (10%). *P <* *0.05. Treatment with placebo from week 0 to week 4 did not result in statistically significant improvements in these six POMS factors.

Figure 3 shows study participants' reports of level of tension, depression, anger, vigor, fatigue, and confusion as rated using the POMS before and after treatment with WGP and placebo. When compared with mean pretreatment values for Wellmune WGP, treatment with WGP for 4 weeks reduced tension by 34%: 7.3 versus 4.8, *P *<* *0.05; depression by 45%: 5.1 versus 2.8, *P *<* *0.05; anger by 41%: 6.6 versus 3.9, *P *<* *0.05; fatigue by 38%: 6.8 versus 5.4, *P *<* *0.05; and confusion by 40%: 5.3 versus 3.2, *P *<* *0.05. Subjects reported increased vigor after WGP treatment compared with before treatment (+10%, WGP pretreatment = 18.3 vs. WGP post-treatment = 20.3). There were no significant differences between the placebo pre-and post-treatment groups or between the WGP post-treatment group and placebo post-treatment in tension, depression, fatigue, confusion, and vigor.

### Global Mood State (cumulative POMS measurement)

**Figure 4:**
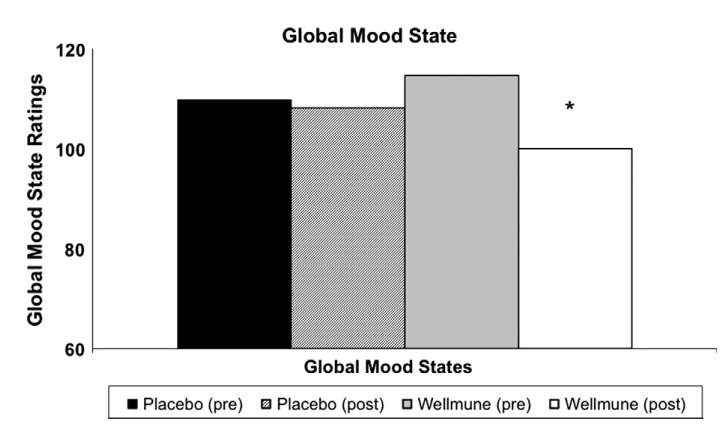
Global Mood States. The Global Mood State showed a 34% improvement (*P *<* *0.05) following 4 weeks of treatment with Wellmune WGP. The placebo group did not show a significant improvement over the 4-week treatment period. A lower number is a “better” global mood state.

Figure 4 compares study participants' reporting of Global Mood State, a cumulative POMS measurement, pre-and post-4-week treatment with WGP or placebo. Global Mood State ratings were 110 and 108 pre-and post-treatment with placebo (n.s.), and 115 and 100 pre-and post-treatment with WGP (13%) for 4 weeks (*P *<* *0.05). This demonstrates that subjects on Wellmune WGP had an improved Global Mood State over the 4-week period compared with WGP pretreatment levels.

### RAND SF-36 (Medical Outcomes Study)

**Figure 5:**
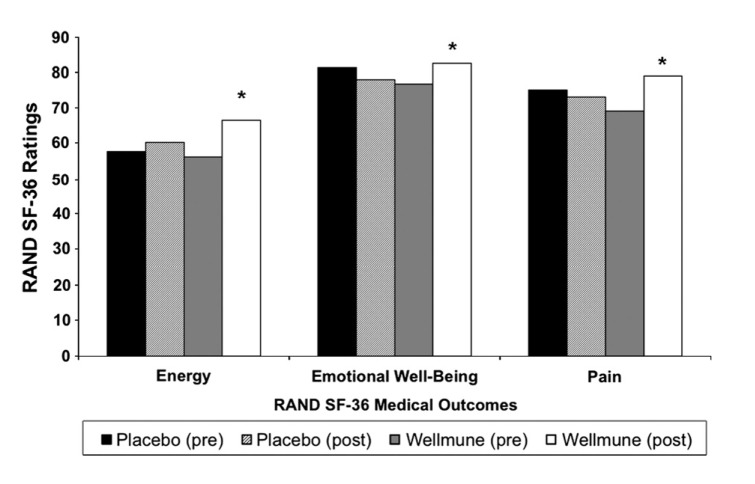
RAND SF-36 Medical Outcomes. After 4 weeks of treatment with Wellmune WGP®, there was an elevation in physical health (11%), energy (19%), and emotional well-being (7%), compared with placebo. *P <* *0.05 compared with placebo.

Figure 5 compares study participants' ratings of physical health, energy, and emotional well-being using the RAND SF-36 (Medical Outcomes Study) before and after treatment with WGP or placebo. After 4 weeks of consuming WGP, there was an increase in study participants' ratings of their energy (+19%, at baseline = 56.1, after treatment = 66.5; *P *≤ 0.05) and emotional well-being (+7%, at baseline = 76.9, after treatment = 82.6; *P *=* *0.034), and reduced rating of pain (−15%, at baseline = 69.1, after treatment = 79.2; *P *<* *0.05). There was a nonsignificant effect of WGP on social functioning, limitations due to emotional problems, and general health, and a trend toward significance on limitations due to physical health(+11%, WGP at baseline = 81.3, WGP after treatment = 90.6; *P *=* *0.06).

### Rhinoconjunctivitis QOL questionnaire

**Figure 6:**
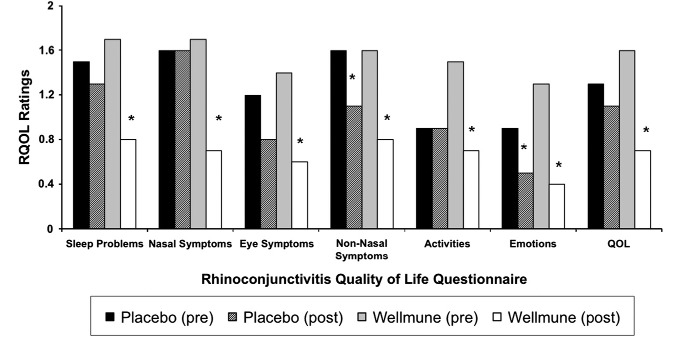
Rhinoconjunctivitis Quality of Life. After 4 weeks of treatment with Wellmune WGP®, there was a reduction in sleep problems (53%), nasal symptoms (59%), and eye symptoms (57%) and an elevation in QOL (56%); *P <* *0.05 compared with placebo.

Figure 6 compares study participants' reporting of sleep problems, nasal and eye symptoms, non-nasal symptoms, practical problems, activities, emotions, and QOL (mean of all items) in the placebo and WGP groups as reported on the Rhinoconjunctivitis QOL questionnaire. In the WGP group, there was a reduction in sleep problems after treatment compared with the ratings at baseline (53%, 0.8 vs. 1.7; *P *<* *0.05), nasal symptoms (59%, 0.7 vs. 1.7; *P *<* *0.0001), eye symptoms (57%, 0.6 vs. 1.4; *P *<* *0.001). Study participants also reported improvements in non-nasal symptoms after treatment with WGP compared with at baseline (50%, 0.8 vs. 1.6; *P *<* *0.01), activities (53%, 0.7 vs. 1.5; *P *<* *0.05), emotions (57%, 0.4 vs. 1.3; *P *<* *0.01). Treatment with placebo also elicited significant decreases in ratings for non-nasal symptoms compared with baseline ratings (31%, 1.1 vs. 1.6; *P *<* *0.05) and emotions (44%, 0.5 vs. 0.9; *P *<* *0.04), but this was not the case for activities (100%, 0.9 vs. 0.9; n.s.).

## Discussion

In this study, we showed that administration of WGP during allergy season reduced the total number of allergy symptoms, rating of symptom severity, and symptom rating on the VAS, but had no effect on IgE concentration compared with placebo treatment in self-described ragweed allergy sufferers. Compared with pretreatment, WGP decreased study participants' ratings of tension, depression, anger, fatigue, and confusion and increased vigor as measured by the POMS (*P *<* *0.05 for all). WGP improved global mood states (POMS) and had an improved effect on physical health, energy, and emotional well-being according to the SF-36 questionnaire, which has been validated for internal consistency, test–retest and interobserver reliability, discriminative properties, and evaluative properties (McHorney and Ware 1994; Leplege et al. 1995). In addition, study participants reported a decrease in sleep problems, nasal and eye symptoms, and QOL in the placebo and WGP treatment groups as reported using the Rhinoconjunctivitis QOL questionnaire.

The potential usefulness of β-glucan as a food additive is supported by work showing that yeast β-glucan exhibits immune-modulating function (Ikewaki et al. 2007); however, our study is one of few that have investigated whether β-glucan can alleviate the symptoms of allergic disease. Yamada et al. (2007) demonstrated a beneficial effect of oral consumption of β-1,3-glucan, derived from shiitake mushroom (*Lentinus edoses* Berk [Sing]), on symptoms of rhinoconjunctivitis and observed that the size of β-glucan particle influences efficacy. Their randomized, placebo-controlled, double-blind study showed that oral administration of superfine dispersed β-1,3-glucan (SDG) for 8 weeks improved ongoing symptoms of rhinoconjunctivitis and rhinitis in Japanese individuals with seasonal allergy to cedar pollen and perennial allergy symptoms (*P *<* *0.0002), compared with an identical amount of nondispersed β-1,3-glucan (NDG) that was administered during the same period to a different group of study participants (Yamada et al. 2007). SDG, with a particle size of 0.08 *μ*m, is thought to be absorbed easily by the intestinal mucosa, in contrast to NDG, which has a particle size of 288 *μ*m. SDG reduced symptoms during the 8-week follow-up period that occurred immediately after the intervention (*P *<* *0.0001) and prevented symptom development when taken prior to symptom onset (*P *<* *0.05). The study also showed that consumption of SDG decreased allergen-specific and total IgE titers (initial high-IgE group, *P *<* *0.043; initial low-IgE group, *P *<* *0.03) and exhibited a good correlation with allergen-specific and total IgE titers (Yamada et al. 2007). Caveats of this study include a short observation period and a small study population.

Similarly, Szabo et al. (2000) showed that β-glucan alleviated some symptoms of allergic conjunctivitis, Sarinho et al. (2009) showed the effect of β-glucan on elevating IL-10 production and relieving asthma symptoms, and Damiani et al. (2011) showed the combination of colloidal silver and β-glucan on viral rhinitis and upper respiratory symptoms in children.

Other studies have also reported no increase or a small increase in Ig concentrations after β-glucan consumption. Lehne et al. (2005) reported no increase in IgA or IgG in serum or saliva after healthy subjects, age 20–30 years, orally consumed 100 mg/day yeast β-glucan for four consecutive days compared to concentrations at baseline. Consumption of 400 mg/day β-glucan for the same amount of time elicited an increase only in salivary IgA (mean ± SD = day 1: 39.6 ± 21.1 vs. 105.4 ± 73.9, *P *<* *0.05). Browder et al. (1990) observed an increase in serum IL-1 in trauma patients, ages 18–65, 3 days after they had been treated with β-glucan (143.4 ± 193% vs. 78.6 ± 11.7%; *P *<* *0.05), but there was no difference after that time point. β-Glucan had no effect on TNFα concentrations.

Allergic rhinitis is an IgE-mediated disease that presents with elevated allergen-specific IgE titers, IgE-dependent activations of mast cells, recruitment of activated eosinophils and T cells to mucosal surfaces, inflammation, and disease (Holgate 1999; Kay 2001a,b) T helper (Th) 2 cells contribute to the induction of IgE-mediated disease by overproducing Th2 cytokines (IL-4, IL-5, and IL-13) at the inflammation site (Romagnani et al. 1991; Huang et al. 1995). However, Th-1 cytokines (IL-12 and IFN-γ) inhibit the Th2 immune response. In a randomized, placebo-controlled, double-blind study of 24 subjects with seasonal allergic rhinitis, Kirmaz et al. (2005) investigated whether β-glucan from *S. cerevisiae* reversed the Th2-mediated immune response in individuals who were sensitized to *Olea europea*. Compared with pretreatment values, 20 mg β-glucan administered for 12 weeks reduced IL-4 (mean ± SEM = 5.48 ± 0.92 vs. 3.66 ± 0.64 pg/mL, *P *=* *0.027) and IL-5 concentrations (mean ± SEM = 8.58 ± 1.58 vs. 5.81 ± 0.83 pg/mL, *P *=* *0.04). Although there was no difference between the placebo group (*n *=* *12) and the β-glucan group (*n *=* *12) in the percentage of eosinophils in nasal lavage fluid after nasal provocation with *Olea europea*, the percentage of eosinophils in nasal lavage fluid was significantly reduced after a nasal provocation test with *Olea europea* compared with concentrations measured before the nasal provocation test (*P *=* *0.01). In contrast, IL-12 levels increased after β-glucan treatment compared with pretreatment values (mean ± SEM = 11.08 ± 2.43 vs. 17.31 ± 2.75 pg/mL, *P *=* *0.008). There was no change in IFN-γ (mean ± SEM = 6.19 ± 1.18 vs. 7.83 ± 1.22 pg/mL, *P *=* *0.1) in the β-glucan group, and no changes in any cytokines for the placebo group. Furthermore, the percentage of eosinophils was unchanged for both treatment groups. The authors proposed that β-glucan is potentially an adjunct to standard therapy in individuals with allergic rhinitis.

Human studies have reported that β-glucan can improve QOL for individuals with cancer or upper respiratory influenza infections. In a randomized, placebo-controlled, double-blind, parallel group intervention study, Feldman et al. (2009) reported an improvement from baseline indicated by scores on two QOL measures of the SF36v-2 questionnaire after study participants, aged 18–65 years, consumed 500 mg/day WGP for 90 days as compared with consuming a rice flour placebo for the same amount of time. Study participants who consumed WGP reported an improved Physical Component Summary score (57.5 ± 4.5 vs. 55.5 ± 3.5; *P *=* *0.029) and a greater General Health summary score (58.7 ± 7.0 vs. 52.0 ± 14.6; *P *<* *0.038) compared with study participants who consumed the placebo. The β-glucan, lentinan, improved QOL in individuals with advanced cancer when administered intravenously (Yoshino et al. 2010) or orally for gastric cancer (Nakano et al. 1999; Kataoka et al. 2009), and orally for pancreatic (Shimizu et al. 2009) and colorectal cancer (Hazama et al. 2009). Nakano et al. (1999) reported that the total QOL score for gastric cancer subjects, in particular appetite and sleep quality, improved with lentinan consumption. In another study of 20 individuals between the ages of 38 and 84 years with advanced malignancies who were undergoing chemotherapy, β-1,3-glucan consumption resulted in a sense of well-being for 60% of study participants, and less fatigue during chemotherapy was reported by 40% of subjects who reported being fatigued (Weitberg 2008).

Immunotherapy has also been used to decrease the effects of ragweed allergy. A randomized, double-blind, placebo-controlled study, investigated the effectiveness of immunotherapy with a Ragweed-Toll-like receptor 9 agonist vaccine for allergic rhinitis (Creticos et al. 2006). Vaccination with the Ragweed-Toll-like receptor 9 agonist did not change the vascular permeability response to nasal provocation in the first season, as reflected in the albumin level in nasal lavage fluid, but improved peak-season rhinitis scores (*P *=* *0.006) on the visual analog score, peak-season daily nasal symptom diary scores (*P *=* *0.02), and mid-season overall quality-of-life scores (*P *=* *0.05). The vaccine also inhibited the seasonal elevation of Amb a 1-specific IgE antibody. In a later ragweed season, compared with placebo, there were improvements in the peak-season rhinitis visual analog score (*P *=* *0.02) and the peak-season daily nasal symptom diary score (*P *=* *0.02). As with our study, the authors noted no change in IgE antibody titer during ragweed season (*P *=* *0.19).

Some studies have reported that airway exposure to β-(1,3)-d-glucan, found in house dust, indoor molds, and some bacteria, can increase the airway allergic response as well as induce other health effects (Rylander et al. 1998). As summarized in Douwes (Douwes 2005), these data are inconsistent and further investigation is necessary to determine whether inhalation of β-(1,3)-d-glucan affects respiratory health. Infants who are exposed to a high concentration of β-(1,3)-d-glucan (60 *μ*g/g) have a reduced risk of recurrent wheezing (adjusted odds ratio = 0.39, 95% CI = 0.16–1.93) and recurrent wheezing combined with allergen sensitization (adjusted odds ratio = 0.57, 95% CI = 0.30–1.10) (Iossifova et al. 2007).

One limitation of our study is the small study population. Additional studies including more study participants should be conducted. Furthermore, ragweed pollen levels were not measured during the study. In addition, there was inadequate characterization of the ragweed allergy status of study participants. Our study involved participants who were self-described allergy sufferers and had not received a clinical diagnosis of ragweed allergy. In contrast, Juniper et al. (1992) characterized subjects' ragweed allergy by requesting a 2-year history of symptoms for which treatment was necessary during the ragweed pollen season and required a positive skin-test result to ragweed pollen extract as a criterion for inclusion in the study.

Our findings suggest that the β-1,3/1,6-glucan, WGP, can be used to decrease the symptoms of allergy in individuals with self-described ragweed allergy. *β*-Glucans can be given orally, are typically less costly than immunotherapies, and have fewer side effects than immunotherapies (Murphy et al. 2010). Furthermore, they occur naturally, and are effective. Additional studies investigating the effect of β-1,3/1,6-glucan on allergies should be conducted.

## References

[b1] Adachi Y, Okazaki M, Ohno N, Yadomae T (1994). Enhancement of cytokine production by macrophages stimulated with (1–>3)-b-D-glucan, grifolan (GRN), isolated from *Grifola frondosa*. Biol. Pharm. Bull..

[b2] Ahren IL, Williams DL, Rice PJ, Forsgren A, Riesbeck K (2001). The importance of a b-glucan receptor in the nonopsonic entry of nontypeable Haemophilus influenzae into human monocytic and epithelial cells. J. Infect. Dis..

[b3] Akramiene D, Kondrotas A, Didziapetriene J, Kevelaitis E (2007). Effects of beta-glucans on the immune system. Medicina (Kaunas).

[b4] Arbes Jr SJ, Gergen PJ, Elliott L, Zeldin DC (2005). Prevalences of positive skin test responses to 10 common allergens in the US population: results from the third National Health and Nutrition Examination Survey. J. Allergy Clin. Immunol..

[b5] Austin JB, Kaur B, Anderson HR, Burr M, Harkins LS, Strachan DP (1999). Hay fever, eczema, and wheeze: a nationwide UK study (ISAAC, international study of asthma and allergies in childhood). Arch. Dis. Child..

[b6] Babineau TJ, Hackford A, Kenler A, Bistrian B, Forse RA, Fairchild PG (1994a). A phase II multicenter, double-blind, randomized, placebo-controlled study of three dosages of an immunomodulator (PGG-glucan) in high-risk surgical patients. Arch. Surg..

[b7] Babineau TJ, Marcello P, Swails W, Kenler A, Bistrian B, Forse RA (1994b). Randomized phase I/II trial of a macrophage-specific immunomodulator (PGG-glucan) in high-risk surgical patients. Ann. Surg..

[b8] Bell S, Goldman VM, Bistrian BR, Arnold AH, Ostroff G, Forse RA (1999). Effect of β-glucan from oats and yeast on serum lipids. Crit. Rev. Food Sci. Nutr..

[b9] Biagini RE, MacKenzie BA, Sammons DL, Smith JP, Krieg EF, Robertson SA (2006). Latex specific IgE: performance characteristics of the IMMULITE 2000 3gAllergy assay compared with skin testing. Ann. Allergy Asthma Immunol..

[b10] Boulet LP, Turcotte H, Laprise C, Lavertu C, Bedard PM, Lavoie A (1997). Comparative degree and type of sensitization to common indoor and outdoor allergens in subjects with allergic rhinitis and/or asthma. Clin. Exp. Allergy.

[b11] Bousquet J, Dhivert H, Michel FB (1994). Current trends in the management of allergic diseases. Allergy.

[b12] Bousquet J, vanCauwenberge P, Khaltaev N (2001). Allergic rhinitis and its impact on asthma. J. Allergy Clin. Immunol..

[b13] Bousquet PJ, Combescure C, Neukirch F, Klossek JM, Mechin H, Daures JP (2007). Visual analog scales can assess the severity of rhinitis graded according to ARIA guidelines. Allergy.

[b14] Browder W, Williams D, Pretus H, Olivero G, Enrichens F, Mao P (1990). Beneficial effect of enhanced macrophage function in the trauma patient. Ann. Surg..

[b15] Brown GD (2006). Dectin-1: a signalling non-TLR pattern-recognition receptor. Nat. Rev. Immunol..

[b16] Brown GD, Gordon S (2001). Immune recognition. A new receptor for b-glucans. Nature.

[b17] Brown GD, Gordon S (2005). Immune recognition of fungal β-glucans. Cell. Microbiol..

[b18] Brown GD, Taylor PR, Reid DM, Willment JA, Williams DL, Martinez-Pomares L (2002). Dectin-1 is a major b-glucan receptor on macrophages. J. Exp. Med..

[b19] Brown GD, Herre J, Williams DL, Willment JA, Marshall AS, Gordon S (2003). Dectin-1 mediates the biological effects of β-glucans. J. Exp. Med..

[b20] Bukantz SC, Lockey RF (2004). Adverse effects and fatalities associated with subcutaneous allergen immunotherapy. Clin. Allergy Immunol..

[b21] Chen J, Seviour R (2007). Medicinal importance of fungal b-(1–>3), (1–>6)-glucans. Mycol. Res..

[b22] Cheung NK, Modak S, Vickers A, Knuckles B (2002). Orally administered b-glucans enhance anti-tumor effects of monoclonal antibodies. Cancer Immunol. Immunother..

[b23] Creticos PS, Schroeder JT, Hamilton RG, Balcer-Whaley SL, Khattignavong AP, Lindblad R (2006). Immunotherapy with a ragweed-toll-like receptor 9 agonist vaccine for allergic rhinitis. N. Engl. J. Med..

[b24] Damiani V, Di CM, Grappasonni G, Di DR, Dominici P (2011). Efficacy of a new medical device based on colloidal silver and carbossimetyl beta glucan in treatment of upper airways disease in children. Minerva Pediatr..

[b25] Delatte SJ, Evans J, Hebra A, Adamson W, Othersen HB, Tagge EP (2001). Effectiveness of b-glucan collagen for treatment of partial-thickness burns in children. J. Pediatr. Surg..

[b26] Dellinger EP, Babineau TJ, Bleicher P, Kaiser AB, Seibert GB, Postier RG (1999). Effect of PGG-glucan on the rate of serious postoperative infection or death observed after high-risk gastrointestinal operations. Betafectin Gastrointestinal Study Group. Arch. Surg..

[b27] Di Carlo FJ, Fiore JV (1958). On the composition of zymosan. Science.

[b28] Douwes J (2005). (1–>3)-b-*D*-glucans and respiratory health: a review of the scientific evidence. Indoor Air.

[b29] Driscoll M, Hansen R, Ding C, Cramer DE, Yan J (2009). Therapeutic potential of various b-glucan sources in conjunction with anti-tumor monoclonal antibody in cancer therapy. Cancer Biol. Ther..

[b30] Du Buske LM, Ling CJ, Sheffer AL (1992). Special problems regarding allergen immunotherapy. Immunol. Allergy Clin. North Am..

[b31] Feldman S, Schwartz HI, Kalman DS, Mayers A, Kohrman HM, Clemens R (2009). Randomized phase II clinical trials of Wellmune WGP® for immune support during cold and flu season. J. Appl. Res..

[b32] de Felippe JJ, SilvaJunior daRochae, Maciel FM, Soares AM, Mendes NF (1993). Infection prevention in patients with severe multiple trauma with the immunomodulator b 1–3 polyglucose (glucan). Surg. Gynecol. Obstet..

[b33] Frenz DA, Palmer MA, Hokanson JM, Scamehorn RT (1995). Seasonal characteristics of ragweed pollen dispersal in the United States. Ann. Allergy Asthma Immunol..

[b35] Hahn PY, Evans SE, Kottom TJ, Standing JE, Pagano RE, Limper AH (2003). *Pneumocystis carinii* cell wall b-glucan induces release of macrophage inflammatory protein-2 from alveolar epithelial cells via a lactosylceramide-mediated mechanism. J. Biol. Chem..

[b36] Hallen H, Djupesland P, Kramer J, Toll K, Graf P (2001). Evaluation of a new method for assessing symptoms. ORL J. Otorhinolaryngol. Relat. Spec..

[b37] Hays RD, Sherbourne CD, Mazel RM (1993). 36-Item Health Survey 1.0. Health Econ..

[b38] Hazama S, Watanabe S, Ohashi M, Yagi M, Suzuki M, Matsuda K (2009). Efficacy of orally administered superfine dispersed lentinan (b-1,3-glucan) for the treatment of advanced colorectal cancer. Anticancer Res..

[b39] Holgate ST (1999). The epidemic of allergy and asthma. Nature.

[b40] Hong F, Yan J, Baran JT, Allendorf DJ, Hansen RD, Ostroff GR (2004). Mechanism by which orally administered b-1,3-glucans enhance the tumoricidal activity of antitumor monoclonal antibodies in murine tumor models. J. Immunol..

[b41] Hornblow AR, Kidson MA (1976). The visual analogue scale for anxiety: a validation study. Aust. N. Z. J. Psychiatry.

[b42] Huang SK, Xiao HQ, Kleine-Tebbe J, Paciotti G, Marsh DG, Lichtenstein LM (1995). IL-13 expression at the sites of allergen challenge in patients with asthma. J. Immunol..

[b43] Ikewaki N, Fujii N, Onaka T, Ikewaki S, Inoko H (2007). Immunological actions of Sophy b-glucan (b-1,3–1,6 glucan), currently available commercially as a health food supplement. Microbiol. Immunol..

[b44] (2008). GRAS Notice for Yeast Beta-Glucan [GRN 239].

[b45] Iossifova YY, Reponen T, Bernstein DI, Levin L, Kalra H, Campo P (2007). House dust (1–3)-b-D-glucan and wheezing in infants. Allergy.

[b46] Juniper EF, Guyatt GH (1991). Development and testing of a new measure of health status for clinical trials in rhinoconjunctivitis. Clin. Exp. Allergy.

[b47] Juniper EF, Willms DG, Guyatt GH, Ferrie PJ (1992). Aqueous beclomethasone dipropionate nasal spray in the treatment of seasonal (ragweed) rhinitis. CMAJ.

[b48] Juniper EF, Guyatt GH, Griffith LE, Ferrie PJ (1996). Interpretation of rhinoconjunctivitis quality of life questionnaire data. J. Allergy Clin. Immunol..

[b49] Kataoka H, Shimura T, Mizoshita T, Kubota E, Mori Y, Mizushima T (2009). Lentinan with S-1 and paclitaxel for gastric cancer chemotherapy improve patient quality of life. Hepatogastroenterology.

[b50] Kay AB (2001a). Allergy and allergic diseases. First of two parts. N. Engl. J. Med..

[b51] Kay AB (2001b). Allergy and allergic diseases. Second of two parts. N. Engl. J. Med..

[b52] Kim HS, Hong JT, Kim Y, Han SB (2011). Stimulatory effect of b-glucans on immune cells. Immune Netw..

[b53] Kirmaz C, Bayrak P, Yilmaz O, Yuksel H (2005). Effects of glucan treatment on the Th1/Th2 balance in patients with allergic rhinitis: a double-blind placebo-controlled study. Eur. Cytokine Netw..

[b54] Kougias P, Wei D, Rice PJ, Ensley HE, Kalbfleisch J, Williams DL (2001). Normal human fibroblasts express pattern recognition receptors for fungal (1–>3)-b-D-glucans. Infect. Immun..

[b55] Kurashige S, Akuzawa Y, Endo F (1997). Effects of *Lentinus edodes*,*Grifola frondosa**Pleurotus ostreatus* administration on cancer outbreak, and activities of macrophages and lymphocytes in mice treated with a carcinogen, N-butyl-N-butanolnitrosoamine. Immunopharmacol. Immunotoxicol..

[b56] Ladanyi A, Timar J, Lapis K (1993). Effect of lentinan on macrophage cytotoxicity against metastatic tumor cells. Cancer Immunol. Immunother..

[b57] Lebron F, Vassallo R, Puri V, Limper AH (2003). *Pneumocystis carinii* cell wall b-glucans initiate macrophage inflammatory responses through NF-kB activation. J. Biol. Chem..

[b58] Lehne G, Haneberg B, Gaustad P, Johansen PW, Preus H, Abrahamsen TG (2005). Oral administration of a new soluble branched b-1,3-D-glucan is well tolerated and can lead to increased salivary concentrations of immunoglobulin A in healthy volunteers. Clin. Exp. Immunol..

[b59] Leplege A, Mesbah M, Marquis P (1995). Preliminary analysis of the psychometric properties of the French version of an international questionnaire measuring the quality of life: the MOS SF-36 (version 1.1). Rev. Epidemiol. Sante Publique.

[b60] Leynaert B, Neukirch C, Liard R, Bousquet J, Neukirch F (2000). Quality of life in allergic rhinitis and asthma. A population-based study of young adults. Am. J. Respir. Crit. Care Med..

[b61] Li B, Allendorf DJ, Hansen R, Marroquin J, Ding C, Cramer DE (2006). Yeast beta-glucan amplifies phagocyte killing of iC3b-opsonized tumor cells via complement receptor 3-Syk-phosphatidylinositol 3-kinase pathway. J Immunol.

[b62] Linder A (1988). Symptom scores as measures of the severity of rhinitis. Clin Allergy.

[b63] Lockey RF, Benedict LM, Turkeltaub PC, Bukantz SC (1987). Fatalities from immunotherapy (IT) and skin testing (ST). J. Allergy Clin. Immunol..

[b64] Lowe EP, Wei D, Rice PJ, Li C, Kalbfleisch J, Browder IW (2002). Human vascular endothelial cells express pattern recognition receptors for fungal glucans which stimulates nuclear factor kB activation and interleukin 8 production. Winner of the Best Paper Award from the Gold Medal Forum. Am. Surg..

[b65] McCann F, Carmona E, Puri V, Pagano RE, Limper AH (2005). Macrophage internalization of fungal b-glucans is not necessary for initiation of related inflammatory responses. Infect. Immun..

[b66] McHorney CA, Jr Ware JE (1994). Lu JF, Sherbourne CD. The MOS 36-item Short-Form Health Survey (SF-36): III. Tests of data quality, scaling assumptions, and reliability across diverse patient groups. Med. Care.

[b67] McNair D, Loor M, Droppleman L (1971). Manual for the profile of mood states.

[b68] McNair D, Heuchert J, Shilony E (2003). http//www.mhs.com.

[b69] Meltzer EO (2001). Quality of life in adults and children with allergic rhinitis. J. Allergy Clin. Immunol..

[b70] Murphy EA, Davis JM, Carmichael MD (2010). Immune modulating effects of β-glucan. Curr. Opin. Clin. Nutr. Metab. Care.

[b71] Nakano H, Namatame K, Nemoto H, Motohashi H, Nishiyama K, Kumada K (1999). A multi-institutional prospective study of lentinan in advanced gastric cancer patients with unresectable and recurrent diseases: effect on prolongation of survival and improvement of quality of life. Kanagawa Lentinan Research Group. Hepatogastroenterology.

[b72] Nathan RA (2003). Pharmacotherapy for allergic rhinitis: a critical review of leukotriene receptor antagonists compared with other treatments. Ann. Allergy Asthma Immunol..

[b73] Nathan RA (2007). The burden of allergic rhinitis. Allergy Asthma Proc..

[b74] (2003). Airborne allergens: something in the air.

[b75] Nicolosi R, Bell SJ, Bistrian BR, Greenberg I, Forse RA, Blackburn GL (1999). Plasma lipid changes after supplementation with b-glucan fiber from yeast. Am. J. Clin. Nutr..

[b76] Oswalt ML, Marshall GD (2008). Ragweed as an example of worldwide allergen expansion. Allergy Asthma Clin. Immunol..

[b77] Pawankar R, Mori S, Ozu C, Kimura S (2011). Overview on the pathomechanisms of allergic rhinitis. Asia Pac. Allergy.

[b78] Pearson A, Lux A, Krieger M (1995). Expression cloning of dSR-CI, a class C macrophage-specific scavenger receptor from *Drosophila melanogaster*. Proc. Natl. Acad. Sci. U.S.A..

[b79] Pongdee T (2011). Ragweed plants packed with pollen. http://www.aaaai.org/Aaaai/media/MediaLibrary/PDF%20Documents/Libraries/EL-ragweed-patient.pdf.

[b80] Price DD, McGrath PA, Rafii A, Buckingham B (1983). The validation of visual analogue scales as ratio scale measures for chronic and experimental pain. Pain.

[b81] Ramakers JD, Volman JJ, Biorklund M, Onning G, Mensink RP, Plat J (2007). Fecal water from ileostomic patients consuming oat β-glucan enhances immune responses in enterocytes. Mol. Nutr. Food Res..

[b82] Rice PJ, Kelley JL, Kogan G, Ensley HE, Kalbfleisch JH, Browder IW (2002). Human monocyte scavenger receptors are pattern recognition receptors for (1–>3)-beta-D-glucans. J. Leukoc. Biol..

[b83] Rice PJ, Adams EL, Ozment-Skelton T, Gonzalez AJ, Goldman MP, Lockhart BE (2005). Oral delivery and gastrointestinal absorption of soluble glucans stimulate increased resistance to infectious challenge. J. Pharmacol. Exp. Ther..

[b84] Riggi SJ, Di Luzio NR (1961). Identification of a reticuloendothelial stimulating agent in zymosan. Am. J. Physiol..

[b85] Romagnani S, Maggi E, Parronchi P, Macchia D, Piccinni MP, Ricci M (1991). Increased numbers of Th2-like CD4 +  T cells in target organs and in the allergen-specific repertoire of allergic patients. Possible role of IL-4 produced by non-T cells. Int. Arch. Allergy Appl. Immunol..

[b86] Ross GD, Cain JA, Myones BL, Newman SL, Lachmann PJ (1987). Specificity of membrane complement receptor type three (CR3) for β-glucans. Complement.

[b87] Rylander R, Norrhall M, Engdahl U, Tunsater A, Holt PG (1998). Airways inflammation, atopy, and (1–> 3)-β-D-glucan exposures in two schools. Am. J. Respir. Crit. Care Med..

[b88] Sarinho E, Medeiros D, Schor D, Rego SA, Sales V, Motta ME (2009). Production of interleukin-10 in asthmatic children after β-1-3-glucan. Allergol. Immunopathol. (Madr.).

[b90] Shah VB, Huang Y, Keshwara R, Ozment-Skelton T, Williams DL, Keshvara L (2008). β glucan activates microglia without inducing cytokine production in Dectin-1-dependent manner. J. Immunol..

[b91] Shimizu K, Watanabe S, Watanabe S, Matsuda K, Suga T, Nakazawa S (2009). Efficacy of oral administered superfine dispersed lentinan for advanced pancreatic cancer. Hepatogastroenterology.

[b92] 7Suzuki I, Tanaka H, Kinoshita A, Oikawa S, Osawa M, Yadomae T (1990). Effect of orally administered b-glucan on macrophage function in mice. Int. J. Immunopharmacol..

[b93] Szabo Z, Szilasi M, Brugos L, Szanto S, Kovacs I, Szeles M (2000). Differences in the changes of allergen-specific IgE serum levels and the chemiluminescence of peripheral blood phagocytes in patients with allergic rhinoconjunctivitis during the ragweed season. Immunol. Lett..

[b94] Taylor PR, Brown GD, Reid DM, Willment JA, Martinez-Pomares L, Gordon S (2002). The β-glucan receptor, dectin-1, is predominantly expressed on the surface of cells of the monocyte/macrophage and neutrophil lineages. J. Immunol..

[b95] Thornton BP, Vetvicka V, Pitman M, Goldman RC, Ross GD (1996). Analysis of the sugar specificity and molecular location of the b-glucan-binding lectin site of complement receptor type 3 (CD11b/CD18). J. Immunol..

[b96] Tsoni SV, Brown GD (2008). β-Glucans and dectin-1. Ann. N. Y. Acad. Sci..

[b97] Tsukada C, Yokoyama H, Miyaji C, Ishimoto Y, Kawamura H, Abo T (2003). Immunopotentiation of intraepithelial lymphocytes in the intestine by oral administrations of β-glucan. Cell. Immunol..

[b98] (2008). http://www.fda.gov/Food/FoodIngredientsPackaging/GenerallyRecognizedasSafeGRAS/GRASListings/ucm153925.htm.

[b99] Vassallo R, Standing J, Limper AH (1999). β-glucan from *Pneumocystis carinii* stimulates TNF a release from alveolar macrophages. J. Eukaryot. Microbiol..

[b100] Vassallo R, Standing JE, Limper AH (2000). Isolated *Pneumocystis carinii* cell wall glucan provokes lower respiratory tract inflammatory responses. J. Immunol..

[b101] Vetvicka V, Yvin JC (2004). Effects of marine β-1,3 glucan on immune reactions. Int. Immunopharmacol..

[b102] Volman JJ, Ramakers JD, Plat J (2008). Dietary modulation of immune function by β-glucans. Physiol. Behav..

[b103] Ware Jr JE (1992). Sherbourne CD. The MOS 36-item short-form health survey (SF-36). I. Conceptual framework and item selection. Med. Care.

[b104] Weitberg AB (2008). A phase I/II trial of beta-(1,3)/(1,6) D-glucan in the treatment of patients with advanced malignancies receiving chemotherapy. J. Exp. Clin. Cancer Res..

[b105] Willment JA, Marshall AS, Reid DM, Williams DL, Wong SY, Gordon S (2005). The human β-glucan receptor is widely expressed and functionally equivalent to murine Dectin-1 on primary cells. Eur. J. Immunol..

[b106] Wooles WR, Diluzio NR (1963). Reticuloendothelial function and the immune response. Science.

[b107] Wopfner N, Gadermaier G, Egger M, Asero R, Ebner C, Jahn-Schmid B (2005). The spectrum of allergens in ragweed and mugwort pollen. Int. Arch. Allergy Immunol..

[b108] Yamada J, Hamuro J, Hatanaka H, Hamabata K, Kinoshita S (2007). Alleviation of seasonal allergic symptoms with superfine β-1,3-glucan: a randomized study. J. Allergy Clin. Immunol..

[b109] Yoon TJ, Kim TJ, Lee H, Shin KS, Yun YP, Moon WK (2008). Anti-tumor metastatic activity of b-glucan purified from mutated *Saccharomyces cerevisiae*. Int. Immunopharmacol..

[b110] Yoshino S, Watanabe S, Imano M, Suga T, Nakazawa S, Hazama S (2010). Improvement of QOL and prognosis by treatment of superfine dispersed lentinan in patients with advanced gastric cancer. Hepatogastroenterology.

[b111] Young MC (1998). Rhinitis, sinusitis, and polyposis. Allergy Asthma Proc..

[b112] Zanon P, Chiodini E, Berra D (2002). Allergy to ragweed in northern Italy and prevention strategies. Monaldi Arch. Chest Dis..

[b113] Zhang K, Petty HR (1994). Influence of polysaccharides on neutrophil function: specific antagonists suggest a model for cooperative saccharide-associated inhibition of immune complex-triggered superoxide production. J. Cell. Biochem..

[b114] Zimmerman JW, Lindermuth J, Fish PA, Palace GP, Stevenson TT, DeMong DE (1998). A novel carbohydrate-glycosphingolipid interaction between a β-(1–3)-glucan immunomodulator, PGG-glucan, and lactosylceramide of human leukocytes. J. Biol. Chem..

